# Significance of negative cervical cytology and positive HPV in the diagnosis of cervical lesions by colposcopy

**DOI:** 10.1515/med-2024-1051

**Published:** 2024-10-02

**Authors:** Changhong Zhang, Liu Dong, Kejie Liu, Hong Xiao, Hao Si, Xiaoqin Wang, Hui Wang

**Affiliations:** Department of Gynecology, The Affiliated Fuyang People’s Hospital of Anhui Medical University, Fuyang, Anhui Province, China; Department of Gynecology, The Affiliated Fuyang People’s Hospital of Anhui Medical University, No. 63, Luci Street, Yingzhou District, Fuyang, Anhui Province, China

**Keywords:** cervical intraepithelial neoplasia, cytodiagnosis, human papillomavirus infection

## Abstract

**Objective:**

The aim of this study was to investigate the significance of colposcopy in diagnosing cervical lesions when negative cervical cytology is combined with positive human papillomavirus (HPV).

**Methods:**

Overall, 370 patients with cervical epithelial lesions who had negative cervical fluid-based cytology combined with positive HPV results were selected and analysed for severity of cervical lesions and HPV distribution.

**Results:**

Among the patients with cervical lesions, 242 had a single HPV infection, and 128 cases had multiple infections. No significant difference was found between HPV single infection and multiple infections in both groups of patients with cervical lesions (*P* > 0.05). Furthermore, 137 had non-HPV 16 and 18, accounting for 37.30% of all the patients with cervical lesions. Among them, HPV 52, 58, and 33 infections were the most common at 38.69, 30.66 and 29.20%, respectively – significantly higher than other high-risk HPV types (*P* < 0.05).

**Conclusion:**

High-risk HPV testing is crucial in patients with negative cervical fluid-based cytology combined with positive HPV results. Patients with HPV 16 and 18 and those with simple HPV 52, 58, and 33 infections should undergo timely colposcopy.

## 1 Introduction

Cervical squamous intraepithelial lesion (SIL) refers to lesions that occur in the cervical region and are closely associated with invasive cervical cancer [1]. SIL and cervical cancer are strongly linked to persistent infection of high-risk human papillomavirus (HR-HPV) [2]. Different subtypes of HR-HPV are associated with varying risks of cervical cancer and cervical intraepithelial neoplasia (CIN) II or higher cervical lesions [3,4].

Currently, direct referral to colposcopy is recommended for patients who test positive for HPV 16 or 18, regardless of the result of cervical liquid-based cytology (LBC). However, there is no consensus on the clinical management of individuals with negative cervical LBC and non-HPV 16/18 infections. Some studies suggest that a re-evaluation after 1 year of infection may be more appropriate for patients with a single non-HPV 16/18 infection [5]. However, owing to the limitations of cervical LBC, such as its high specificity and low sensitivity compared to histology [6,7], follow-up management for this population may result in missed diagnoses.

Therefore, some researchers believe that patients with persistent non-16/18 HPV infections should undergo colposcopic biopsy to reduce the risk of missing CIN and cancer [8]. The long-term clinical management of these patients remains controversial.

This study analysed the distribution of HPV types in patients with negative cervical LBC and positive HPV results to provide a reference for the clinical management of this population.

## 2 Materials and methods

### 2.1 Clinical data

A retrospective analysis was conducted on 370 patients aged 23–74 years (mean age: 42.72 ± 10.00 years) with cervical epithelial lesions who were treated at our hospital between June 2018 and December 2022. All patients underwent colposcopy examinations and multiple-point biopsies.

The inclusion criteria were negative cervical cytology results, HPV infection and colposcopic biopsy pathology results of CIN or cervical cancer.

In contrast, patients who had undergone hysterectomy or pelvic radiotherapy, those with concomitant malignancies, pregnant patients, and those with concomitant immune diseases were excluded.

The cervical lesions were categorised into low-grade lesions (i.e., CIN I) and high-grade lesions (i.e., CIN II, II-III, and III and cervical cancer) based on severity. Furthermore, based on the number of HPV types detected, the patients were divided into single infections (infection with only one HPV type) and multiple infections (infection with two or more HPV types).

### 2.2 Cervical LBC test

A specialised cervical brush was utilised to collect exfoliated cells from the cervix, encompassing the outer cervix and cervical canal, for cervical LBC testing. Then, the cells were immersed in a vial containing cell preservation liquid (Wuhan Landing Intelligent Medicine Co., Ltd), and the exfoliated cells were preserved. The vials were stored at 25°C and the samples were analysed in the cytosol as soon as possible and within half a month. The corresponding tests were conducted using ThinPrep technology (Hologic).

### 2.3 HPV genotyping test

A specialised HPV cervical brush was gently rotated and swiped on the cervix at the cervical os. Then, the brush was placed in a sterile glass tube containing cell preservation liquid (Jiangsu Kangjian Medical Supplies Co., Ltd). These glass vials were stored at 2–8°C and tested within 3 days.

The Shanghai automated sample preparation system and PCR combined with Taqman technology were used for the qualitative detection and genotyping of 21 HPV types, including high-risk types, such as HPV 16, 18, 31, 33, 35, 39, 45, 51, 52, 53, 56, 58, 59, 66, 68, 26, and 82 and low-risk types, namely, HPV6, 11 and 81 for specific DNA nucleic acid fragments.

### 2.4 Vaginoscopy

The Shenzhen Goldway electronic colposcope was used for examinations conducted 3–7 days after menstruation. The cervix surface and vaginal secretions were gently wiped off with a saline cotton swab. Under the guidance of the colposcope, the cervix and vaginal area were coated with 3% acetic acid. After 1–2 min, the cervical epithelium completely changed in colour. Suspicious lesions were carefully observed and then coated with 5% Lugol’s iodine solution. Vaginoscopy was performed, and if type 3 transformation zones were present, endocervical curettage was conducted. If suspicious lesions were identified, multiple biopsies were taken at the 3, 6, 9 and 12 o’clock positions or at the site of the suspicious lesion. The specimens were divided into containers and preserved in a formalin solution. The results were interpreted by two pathologists.

### 2.5 Observation indicators

The distribution of HPV genotypes was analysed in the groups of patients with cervical lesions.

### 2.6 Statistics and analysis

SPSS 20.0 statistical software was utilised for data analysis. Count data were presented as frequency or percentage, and the rates were compared using the chi-square test. *P* < 0.05 indicated a significant difference.


**Ethical approval:** This study was approved by the hospital’s ethics committee and is in compliance with the Helsinki Declaration.
**Informed consent:** All patients agreed to participate in the study and signed the informed consent forms.

## 3 Results

### 3.1 Distribution of HPV infection types in patients with cervical lesions

The distribution of HPV types in patients can be categorised as either single infection or multiple infections. Among the cases, most were single infections, accounting for 242 cases (65.41%), whereas multiple infections accounted for 128 cases (34.59%). No significant difference was noted in the prevalence of single infection and multiple infections between the two groups of cervical lesion patients (*P* > 0.05), as shown in Table 1.

**Table 1:** HPV type distribution in two cohorts of cervical lesion patients

Classification of HPV infections	Number of cases	Cervical low-grade lesions (*n* = 64)	Cervical high-grade lesions (*n* = 306)	*χ* ^2^	*P*
Monotype	242	38 (59.38)	204 (66.67)	1.244	0.265^#^
Polytype	128	26 (40.62)	102 (33.33)		

Note: ^#^ *P* > 0.05, indicating no statistically significant difference.

Of the cases with single HPV infection, non-HPV 16 and 18 types accounted for 83 cases (34.30%). In the cervical low-grade lesion group, non-HPV 16 and 18 types accounted for 18 cases (47.37%), whereas in the cervical high-grade lesion group, it accounted for 65 cases (31.86%). No significant difference was found between the two groups (*P* > 0.05), as shown in Table 2.

**Table 2:** Proportion of HPV monotypes in different subtypes of cervical lesions (*n*, Percentage: %)

HPV subtype	Cervical low-grade lesions (*n* = 38)	Cervical high-grade lesions (*n* = 204)	c2	*P*
HPV16/18 type	20 (52.63)	139 (68.14)	3.418	0.065^#^
Non-HPV16/18 type	18 (47.37)	65 (31.86)		

Note: ^#^ *P* > 0.05, indicating no statistically significant difference.

In the high-grade cervical lesion group, there were 14 cases of cervical cancer and 11 cases of single infection, accounting for 78.57%, including 6 cases of HPV 16, 3 cases of HPV 18, 1 case of HPV 45, and 1 case of HPV 51 and 3 cases of multiple infections, 1 case was positive for HPV 16 and 52, 1 case was positive for HPV 18 and 52, and 1 case was positive for HPV 33 and 81 (Table 3).

**Table 3:** HPV type distribution in cervix

HPV infections	Number of cases
HPV 16	6
HPV 18	3
HPV 45	1
HPV 16 and 52	1
HPV 18 and 52	1
HPV 33 and 81	1

### 3.2 Distribution of special HR HPV infection status

Among all patients with cervical lesions, 137 were identified with non-HPV 16 and 18 types, accounting for 37.30% of the total. Of these patients, 30 were in the cervical low-grade lesion group and 107 in the cervical high-grade lesion group (Table 4(a)).

**Table 4:** Distribution of specific HR-HPV infection status (cases, Percentage: %)

HPV type	Cervical low-grade lesions	Cervical high-grade lesions	Total
(a)			
HPV 16/18 type	34	199	233 (62.97)
Non-HPV 16/18 type	30	107	137 (37.30)

HPV type	Cervical low-grade lesions	Cervical high-grade lesions	Case (%)
(b)			
HPV 52	10	43	53 (38.69)
HPV 58	7	35	42 (30.66)
HPV 33	4	36	40 (29.20)
HPV 31	4	15	19 (13.87)^a,b,c^
HPV 39	3	8	11 (8.03)^a,b,c^
HPV 51	2	8	10 (7.30)^a,b,c^
HPV 35	4	4	8 (5.84)^a,b,c^
HPV 56	4	4	8 (5.84)^a,b,c^
HPV 68	2	5	7 (5.20)^a,b,c^
HPV 66	2	4	6 (4.38)^a,b,c^
HPV 53	1	3	4 (2.92)^a,b,c^
HPV 45	1	3	4 (2.92)^a,b,c^
HPV 59	1	2	3 (2.19)^a,b,c^
HPV 82	1	2	3 (2.19)^a,b,c^

Note: ^a^Statistically significant difference compared to HPV 52, *P* > 0.05. ^b^Statistically significant difference compared to HPV 58, *P* > 0.05. ^c^Statistically significant difference compared to HPV 33, *P* > 0.05.

Among the non-HPV 16 and 18 types, HPV 52, 58, and 33 infections were the most prevalent, representing 38.69%, 30.66% and 29.20%, respectively. These percentages significantly exceeded those of other HR-HPV infections and demonstrated significant differences (*P* > 0.05). However, no significant difference was noted among HPV 52, 58, and 33 (Table 4(b)).

## 4 Discussion

In recent decades, the increase and popularisation of cervical cancer precursor lesion screening methods have led to the early detection and treatment of cervical cancer and precursor lesions. However, in China, there are currently several factors contributing to the low sensitivity and high missed diagnosis rate of LBC for cervical lesions. These include a lack of experienced cytology professionals, significant differences in the level of LBC testing between different hospitals and inadequate cell preparation techniques and quality control systems for cytological diagnosis [9].

As more studies have shown the crucial role of HR-HPV genotyping in the screening of cervical cancer precursor lesions and cervical cancer [10], the aim of the present study was to analyse the distribution of HPV infections in patients with cervical lesions to provide insights into their clinical management.

The analysis revealed that HPV infections in patients with cervical lesions were mainly single infections, consistent with a study conducted in Bazhong, Sichuan Province [11]. Furthermore, no significant relationship was found between the severity of cervical lesions and the type of HPV infection (single or multiple infections), which aligns with previous findings indicating that multiple HPV infections do not necessarily increase the risk of further progression of cervical lesions compared to single HPV infection [12]. This may be due to specific combinations of HPV infections having synergistic or antagonistic effects on the risk of cervical lesion progression.

According to current guidelines, patients who test positive for HPV 16/18, regardless of cytology results, should be referred for colposcopy. Moreover, our data support this recommendation, as HPV 16 and 18 infections account for most cervical cancer cases. For patients who test positive for non-HPV 16/18 types and have negative cytology results, it is recommended to retest after 1 year [13,14]. However, owing to patients’ inability to undergo regular follow-up examinations, the risk of cervical lesions continues to increase for women with non-16/18 HR-HPV positivity. Therefore, the management of women who are cytology-negative but non-16/18 HR-HPV-positive remains controversial.

A prospective study involving 165 patients demonstrated that among individuals with pure non-HPV 16/18 infection (negative LBC of the cervix), 13.2% developed cervical lesions upon reassessment after 1 year. However, only two cases were identified as high-grade SIL (HSIL). Thus, the researchers recommend reassessing patients who test positive for non-HPV 16/18 types and have negative cytology results after 1 year [5].

Nonetheless, another study [15] discovered that aside from HPV 16 and 18, other high-risk HPV types exhibit a substantial proportion of cervical lesions. Moreover, because of limitations in the diagnosis of cervical LBC, relying solely on negative cytology results may result in missed diagnoses for some patients with non-16/18 HR-HPV-induced cervical lesions.

Moreover, a study conducted in southern Shanghai revealed a missed diagnosis rate of 62.81% for cervical LBC in patients with non-16/18 HR-HPV infection with HSIL or cancerous lesions, indicating that colposcopy should be performed for persistent non-16/18 HR-HPV infection [8].

Furthermore, our subsequent study showed that among patients with cervical lesions and non-16/18 HR-HPV infection, HPV 52, 58, and 33 exhibited the highest positivity rates and were significantly higher than other HPV types. This finding aligns with a meta-analysis on HPV infection types in the Chinese population [16], which identified HPV 16, 58, 52, 33, and 31 as the most common types among women with CIN II/III. Zhou et al. [17] reported that infection with specific HPV types (31/33/52/56/58/68) increases the risk of high-grade lesions in individuals with pure non-HPV 16/18 infections. Additionally, a study on the global distribution of HPV infection types in invasive cervical cancer patients indicated that, in the Asian population, HPV 52 and 58 were prevalent alongside HPV 16 and 18 [18]. Therefore, for patients with pure HPV 52, 58, or 33 infection, colposcopy is recommended even if the cervical LBC is negative to prevent missed diagnoses. Other HR-HPV-positive women may consider joint screening after 12 months.

The present study primarily investigated the distribution of HPV in patients with cervical cytology-negative cervical lesions and found that in this group of patients, in addition to HPV 16 and 18, a high percentage of HPV 52, 58, and 33 was found. Based on practical clinical work experience, we recommend colposcopy for all patients who are positive for HPV 52, 58, or 33. The possible occurrence of CIN in patients who are positive for HPV 52, 58, and 33 and differences in the pathogenesis of cervical lesions induced by HPV 52, 58, and 33 compared to other HPV infection types should be investigated. This study has included a large and detailed amount of clinical data to analyse and provides valid recommendations for future clinical work. However, because this study was a retrospective analysis, it did not evaluate the prevalence of these specific HPVs leading to cervical lesions in the population; therefore, further studies are required to explore this aspect. Furthermore, it has been shown that the complex intravaginal environment is closely related to cervical intraepithelial lesions and oxidative stress in the vagina may promote SIL progression and cervical cancer [19]. Hence, their pathogenesis should be explored in future studies. In addition, fertility-preserving treatments are critical for young women with early-stage cervical cancer, especially for patients with cancer lesions larger than 2 cm. Currently, the commonly used surgical options are vaginal radical trachelectomy and conization. Fertility preservation is expected to maintain reproductive function in this population; however, standard, well-established treatment options are lacking [20].

In conclusion, HR-HPV genotyping testing plays a crucial role in the diagnosis of cervical lesions in patients with negative cytology results. Colposcopy and biopsy are crucial for timely detection and appropriate treatment of high-grade lesions or invasive cervical cancer, especially in cases where LBC yields false-negative results. To prevent missed diagnoses, women who test positive for HPV 52, 58, or 33 should undergo colposcopy even if their cytology results are negative. Women with other HR-HPV types may consider joint screening after 12 months.

## References

[1] Tainio K, Athanasiou A, Tikkinen KAO, Aaltonen R, Cárdenas J, Hernándes, et al. Clinical course of untreated cervical intraepithelial neoplasia grade 2 under active surveillance: Systematic review and meta-analysis. BMJ. 2018 Feb;360:k499. 10.1136/bmj.k499. PMC5826010 29487049

[2] Sasagawa T, Dong Y, Saijoh K, Satake S, Tateno M, Inoue M. Human papillomavirus infection and risk determinants for squamous intraepithelial lesion and cervical cancer in Japan. Jpn J Cancer Res. 1997 Apr;88(4):376–84. 10.1111/j.1349-7006.1997.tb00271.x. PMC5921426 9197529

[3] Chen L, Dong B, Zhang Q, Mao X, Lin W, Ruan G, et al. HR-HPV viral load quality detection provides more accurate prediction for residual lesions after treatment: a prospective cohort study in patients with high-grade squamous lesions or worse. Med Oncol. 2020;37(5):37. 10.1007/s12032-020-01446-3. 32232578

[4] Pańczyszyn A, Boniewska-Bernacka E, Głąb G. Telomere length in leukocytes and cervical smears of women with high-risk human papillomavirus (HR HPV) infection. Taiwan J Obstet Gynecol. 2020;59(1):51–5. 10.1016/j.tjog.2019.12.013. 32039800

[5] Vargas S, Valente MP, Almeida MM, Neves J, Calhaz-Jorge C. Isolated non-16/18 high-risk human papillomavirus cervical infection: Re-evaluation after one year. Acta Med Port. 2019;32(9):588–92. 10.20344/amp.12146. 31493362

[6] Huang X. Analysis on the application value of HPV genotyping combined with TCT examination in early cervical cancer screening. Contemp Med. 2022;28(2):136–8.

[7] Sun XF, Gu YQ, Wang AC, Wang J, Xie JL. Value assessment of high-risk HPV test and TCT in the screening of cervical carcinoma. Zhonghua Shi Yan He Lin Chuang Bing Du Xue Za Zhi. 2013;27(4):273–6. 24579473

[8] Wang X, Wu S, Li Y. Risks for cervical abnormalities in women with non-16/18 high-risk human papillomavirus infections in south Shanghai, China. J Med Virol. 2021;93(11):6355–61. 10.1002/jmv.26923. 34232523

[9] Cheng JX, Yao LL, Xiang H, Zhan YJ, Zhou P, Yuan M, et al. Cervical cytology ASCUS patients with HPV detection and clinical value. Clin Exp Obstet Gynecol. 2016;43(4):592–6. 10.12891/ceog2545.2016. 29734556

[10] Qin H, Yang G, Xu Y, Zhang H, Zhao J. Research progress on the screening value of HPV E6/E7, cervical liquid-based cytology, and colposcopy biopsy in cervical cancer. Chin J Obstet Gynecol. 2022;23(03):332–4.

[11] Shao W, Wu H, Liu Q, Zhang D. HPV infection status and its influence on the incidence of cervical lesions in women undergoing physical examination in Bazhong Area. Mod Oncol Med. 2022;30(2):286–9.

[12] Del Prete R, Ronga L, Magrone R, Addati G, Abbasciano A, Di Carlo D, et al. Epidemiological evaluation of human papillomavirus genotypes and their associations in multiple infections. Epidemiol Infect. 2019;147:e132. 10.1017/S0950268819000271. PMC6518506 30869020

[13] Chelmow D. Practice Bulletin No. 157: Cervical cancer screening and prevention. Obstet Gynecol. 2016;127(1):e1–20. 10.1097/AOG.0000000000001273. 26695583

[14] Massad LS, Einstein MH, Huh WK, Katki HA, Kinney WK, Schiffman M, et al. Updated consensus guidelines for the management of abnormal cervical cancer screening tests and cancer precursors. J Low Genit Tract Dis. 2013;17(1 Suppl 1):S1–27. 10.1097/LGT.0b013e31826d4e63. 23519301

[15] Myers ER, McCrory DC, Nanda K, Bastian L, Matchar DB. Mathematical model for the natural history of human papillomavirus infection and cervical carcinogenesis. Am J Epidemiol. 2000;151(11):1158–71. 10.1093/aje/151.11.1158. 10905528

[16] Zhang J, Cheng K, Wang Z. Prevalence and distribution of human papillomavirus genotypes in cervical intraepithelial neoplasia in China: A meta-analysis. Arch Gynecol Obstet. 2020;302(6):1329–37. 10.1007/s00404-020-05709-3. PMC7584548 32914222

[17] Zhou H, Xiong A, Liu Q. Necessity of colposcopy screening in women with pure non-HPV16/18 infection in Nanjing and predicting risk outcomes. Chin J Sexol. 2021;30(7):80–3.

[18] Clifford GM, Smith JS, Plummer M, Muñoz N, Franceschi S. Human papillomavirus types in invasive cervical cancer worldwide: A meta-analysis. Br J Cancer. 2003 Jan;88(1):63–73. 10.1038/sj.bjc.6600688. PMC2376782 12556961

[19] Despot A, Fureš R, Despot AM, Mikuš M, Zlopaša G, D’Amato A, et al. Reactive oxygen species within the vaginal space: An additional promoter of cervical intraepithelial neoplasia and uterine cervical cancer development? Open Med (Wars). 2023 Oct;18(1):20230826. 10.1515/med-2023-0826. PMC10590607 37873540

[20] D’Amato A, Riemma G, Agrifoglio V, Chiantera V, Laganà AS, Mikuš M, et al. Reproductive outcomes in young women with early-stage cervical cancer greater than 2 cm undergoing fertility-sparing treatment: A systematic review. Medicina (Kaunas). 2024 Apr;60(4):608. 10.3390/medicina60040608. PMC11051883 38674254

